# Walking and cycling for commuting, leisure and errands: relations with individual characteristics and leisure-time physical activity in a cross-sectional survey (the ACTI-Cités project)

**DOI:** 10.1186/s12966-015-0310-5

**Published:** 2015-12-09

**Authors:** Mehdi Menai, Hélène Charreire, Thierry Feuillet, Paul Salze, Christiane Weber, Christophe Enaux, Valentina A. Andreeva, Serge Hercberg, Julie-Anne Nazare, Camille Perchoux, Chantal Simon, Jean-Michel Oppert

**Affiliations:** Université Paris 13, Equipe de Recherche en Epidémiologie Nutritionnelle, Centre de Recherche en Epidémiologie et Statistiques, Inserm (U1153), Inra (U1125), Cnam, COMUE Sorbonne Paris Cité, Bobigny, F-93017 France; Department of Geography, Lab-Urba, Urbanism Institute of Paris, Paris-Est Créteil University, Paris, France; Laboratoire Image, Ville et Environnement, Université de Strasbourg, Strasbourg, France; Department of Public Health, Hôpital Avicenne (AP-HP), Bobigny, France; CARMEN, Inserm U1060, Université de Lyon 1, Inra U1235, Lyon, France; Department of Nutrition Pitié-Salpêtrière Hospital (AP-HP), Institute of Cardiometabolism and Nutrition (ICAN), Université Pierre et Marie Curie-Paris 6, Paris, France

**Keywords:** Walking, Cycling, Active transport, Physical activity, Correlates, Age, Cross-sectional, Web-cohort, Commuting, Leisure, Errands

## Abstract

**Background:**

Increasing active transport behavior (walking, cycling) throughout the life-course is a key element of physical activity promotion for health. There is, however, a need to better understand the correlates of specific domains of walking and cycling to identify more precisely at-risk populations for public health interventions. In addition, current knowledge of interactions between domains of walking and cycling remains limited.

**Methods:**

We assessed past-month self-reported time spent walking and cycling in three specific domains (commuting, leisure and errands) in 39,295 French adult participants (76.5 % women) of the on-going NutriNet Santé web-cohort. Multivariate logistic regression models were used to investigate the associations with socio-demographic and physical activity correlates.

**Results:**

Having a transit pass was strongly positively associated with walking for commuting and for errands but was unrelated to walking for leisure or to all domains of cycling. Having a parking space at work was strongly negatively associated with walking for commuting and cycling for commuting. BMI was negatively associated with both walking for leisure and errands, and with the three domains of cycling. Leisure-time physical activity was negatively associated with walking for commuting but was positively associated with the two other domains of walking and with cycling (three domains). Walking for commuting was positively associated with the other domains of walking; cycling for commuting was also positively associated with the other domains of cycling. Walking for commuting was not associated with cycling for commuting.

**Conclusions:**

In adults walking and cycling socio-demographic and physical activity correlates differ by domain (commuting, leisure and errands). Better knowledge of relationships between domains should help to develop interventions focusing not only the right population, but also the right behavior.

## Background

Active transportation is now considered as a key element of physical activity promotion for health [1]. Walking and cycling in everyday life may help to achieve sufficient physical activity for health benefits at the population level [2]. Walking and cycling are relatively easy to include in daily routines and have societal benefits such as positive impact on traffic, air pollution, and greenhouse gas emissions [3]. Walking and cycling, like physical activity in general, should be considered as multi-factorial behaviors varying throughout the lifecourse and by domain, such as commuting, leisure or errands [4].

For interventions to attain success in target populations, there is a need to better understand the determinants of adoption and maintenance of walking and cycling [5]. Correlates of active travel include personal, social and environmental factors [6]. There is evidence that gender and having access to a car or being overweight are associated with active travel [7–9]. For age, mixed findings have been reported with null [10, 11] or negative [11–14] associations with walking or cycling. One possible reason for these inconsistent findings may be related to the fact that very few studies have specifically assessed correlates of walking and cycling by domain, i.e. the different contexts of daily life where physical activity takes place (commuting, leisure, and work). Knowledge of domains may help to design interventions and guide public health policies to target at-risk populations.

To date, some studies have explored associations with other types of physical activity such as leisure-time physical activity (LTPA) and overall active transportation [11, 15–17], walking [13, 18–21] or cycling [13, 19–22]. For example, in a large cross-sectional survey of 127,610 Canadian adults, Butler et al. found a positive association between walking for transportation (to work, school and errands) and LTPA, and an even stronger association for cycling [13]. Sahlqvist et al. found recently in the iConnect study that a 1-year decrease in cycling for commuting (not for walking) was associated with a decrease in LTPA [19]. A limitation in previous literature is related to heterogeneity in the definition of active transportation variables. This underscores the need for a much more detailed assessment of walking and cycling by domain, to better understand how walking and cycling are integrated into an overall physically active lifestyle. We hypothesize that 1) there are significant positive interrelations between walking and cycling domains and 2) there are significant positive relations between walking and cycling, on one hand, and LTPA on the other.

Consequently, the objectives of the present cross-sectional study, in a large sample of French adults, were 1) to identify personal and socio-demographic correlates of walking and cycling according to the different domains (commuting, leisure and errands), and 2) to explore the interrelationships of these domains as well as associations with LTPA.

## Methods

### Ethics statement

This study was approved by the "Comité National Informatique et Liberté" (CNIL n°908450, n° 909216 and DR-2012-576). The NutriNet-Santé Study (see below) was approved by the Institutional Review Board of the French Institute for Health and Medical Research (IRB Inserm n°0000388FWA00005831). Written informed consent was obtained from all subjects.

### Study design and participants

We analyzed cross-sectional data from participants in the NutriNet-Santé Study, a web-based prospective observational cohort launched in France in 2009, focusing on the relationship between nutrition and chronic disease risks as well as the determinants of dietary behaviors. Volunteers aged 18 years or older (age range 18–96 years) living in France and having access to the Internet fill in self-administered web-based questionnaires at baseline and then regularly during follow-up using a dedicated, secure website. A detailed description of the NutriNet-Santé study has been published previously [23].

Participants in the present study were subjects from the NutriNet-Santé cohort who completed a questionnaire on physical activity and mobility, administered from February 15 to August 15 2013 (*n* = 55,694; 48.5 % participation rate). This questionnaire was designed to assess active transport in everyday life over the past four weeks.

From the sample who filled in the physical activity questionnaire, 1730 participants were excluded because of physical limitations to mobility, such as self-reported motor impairments (*n* = 927) or self-reported limitations to walking (item ‘Ability to walk 100 m’ *n* = 803). Additionally we excluded participants who were pregnant (*n* = 730), reported implausible physical activity values (*n* = 2817), or had missing data regarding the covariates used in multivariable analyses (*n* = 11,122). Thus, we reached a final sample of 39,295 subjects with a mean ± SD age of 49.1 ± 14.4 years.

### Measures

#### Walking, cycling and other types of physical activity

Habitual physical activity was assessed using a dedicated developed questionnaire, the Sedentary, Transportation and Activity Questionnaire (STAQ). Briefly, the STAQ is based on the Recent Physical Activity Questionnaire (RPAQ) [24], with additional specific items on travel-related activities and sedentary behavior by domain. To assess more precisely transport behaviors (active and passive), subjects were asked to report their travel time for commuting, leisure and errands (defined as non-commuting non-leisure purposes such as shopping, bringing children to school, going to the movies, etc.) for the past 4 weeks.

Physical activity assessment using the RPAQ has been validated against energy expenditure measurements using the doubly-labelled water [24]. The validity and reliability of the specific questions on travel-related activities have been assessed in 88 subjects aged 20–65 years (article under revision). Briefly, the estimate of active transport time was found significantly correlated with data obtained by a logbook (*r* = 0.40, mean bias 7.2 %), and reliability was moderate (intra-class coefficient 0.47 for 1-month test-retest).

The travel questions were detailed by type of transportation (car, public transportation, walking, cycling, and other mechanical vehicle) and included the mean number of days per week and the mean duration per day where the particular type of transportation was used. For each type of transportation, results were expressed in h/week. Walking and cycling by domain were dichotomized (≥0.5 h/week and ≥ 0 h/week, respectively). We chose 0.5 h/week for walking (approximately 5 min/day 6 days/week) to represent a minimum level of walking beyond mandatory steps during daily living at home. When analyses were performed using different thresholds (≥1.0 h/week and ≥ 0.5 h/week for walking and cycling, respectively), similar results were observed (data not shown). There were six outcomes: walking for commuting, walking for leisure, walking for errands, cycling for commuting, cycling for leisure and cycling for errands. For each multivariate model with one of these outcomes, other outcomes were used as covariates. When walking or cycling for commuting was used as covariate, we created three-class variables (e.g. for walking for commuting: do not work/work but do not perform walking for commuting/work and perform walking for commuting); results for the “do not work” class are not presented.

For domestic physical activity, a unique question was asked about the time spent per week usually doing moderate to vigorous activities such as cleaning the floor, using vacuum or similar activity. Based on the median, this variable was dichotomized as ± 7 h per week (i.e. 1 h/day). LTPA was obtained by summing weekly durations of each activity reported in the leisure section. Walking for leisure and cycling for leisure were not included in the calculation because there were part of the walking and cycling variables. The resulting LTPA variable was categorized based on quartiles: less than 1 h per week (1st quartile), between 1 h and 2 h30 per week (quartiles 2), more than 2 h30 per week (quartile 3–4).

### Covariates

Individual and socio-demographic variables were assessed by self-administered questionnaire completed by participants at inclusion. Data included age, gender, weight and height, educational level (more or less than 2 years of university), household income (0–1,430 Euros/month, 1,430–2,330 Euros/month, 2,330–3,780 Euros/month, more than 3,780 Euros/month, do not know/do not want to respond), smoking status (yes or no), household composition (living alone or in a couple), presence of children at home (aged under 13 years, between 14 and 18 years), self-rated health (poor to average, good to very good) and home address. Age was categorized by 5-year age group for figures and used continuously in other analyses. Body mass index (BMI) was calculated as reported weight (kg) divided by reported height squared (m^2^).

Weekly number of working hours was asked during the past 4 weeks and the weekly mean duration was computed. Distance to work was estimated based on the frequency and the duration of each type of transport used for commuting, on the basis of 25 km/h for car, 25 km/h for public transport, 10 km/h for cycling, 4 km/h for walking and 10 km/h for others modes of transportation [25]. The type and amount of physical activity at work was assessed with a 4-category qualitative question [24] (sedentary, standing, manual or heavy manual job) and a binary variable was created (sedentary or standing job, manual and heavy manual job). Parking at work was assessed by a binary variable. Sedentary leisure activities were derived from questions asking participants to report hours per day (excluding working hours) usually spent on an average work/non-work day over the past four weeks – watching television, DVDs or other videos; using a computer, a tablet, or playing screen-based video games. The sum of all the mean durations per week of these activities was categorized as between 0 and ≤ 2 h per day, between 2 h and 4 h per day and more than 4 h per day.

City density (number of inhabitants/surface) was obtained from the Census databases (www.insee.fr) and categorized as follows: 0–300 people per km^2^ (rural area), 300–2000 people per km^2^ and more than 2000 people per km^2^ (high density city).

### Statistical analyses

Continuous variables were summarized by means ± standard deviations (SD) and categorical variables by frequencies. Associations between practice of walking or cycling and potential correlates were assessed using multivariate logistic regression models. Results are expressed as odds ratios (OR) with 95 % confidence intervals (CI). We also computed Nagelkerke's R^2^ for each model. We initially identified potential correlates and covariables in models through bivariate analyses and existing literature. Covariates included age, income, self-rated health status, smoking status, leisure screen time, city density, distance to work, and time spent at work. For all analyses, the significance level was set at 0.05 and all tests were two-tailed. All statistical analyses were performed using SAS software (version 9.3, SAS Institute Inc., Cary, NC, USA).

## Results

### Characteristics of the study population

Compared to subjects included in the NutriNet-Study but not included in the present analyses, our study population comprised more men (23.5 vs. 21.8 %, *p* < 0.001), older subjects (49.1 vs. 43.0 years, *p* < 0.0001), and more subjects with education level of at least 2 years at university (64.3 vs. 57.8 %, *p* < 0.0001). Subjects were mostly middle-aged, with a majority of women, and two-thirds being highly educated (Table 1). Two-thirds of subjects also reported having a job, which was of a sedentary type for a majority of them. Overall, walking for commuting, leisure and errands was performed by 26.3 %, 41.9 % and 42.0 % of subjects, respectively. Cycling for commuting, leisure and errands was performed by 7.2 %, 9.7 % and 8.6 % of subjects, respectively.

**Table 1:** Characteristics of study population

*n* **=** 39,295	Mean (SD) or %
Individual characteristics	
Age (y)	49.1 (14.4)
Gender (men)	23.5
BMI (kg/m^2^)	23.8 (4.3)
Education (≥2 y of university)	64.3
Living with a partner	73.5
Have a child at home under 14y	22.9
Have a child at home between 14y and 18y	11.6
Work and transport related characteristics	
Employed	68.7
Having a public transport pass	19.8
If working, having a sedentary job	90.6
If working, parking place at work	37.7
Walking	
Commuting among workers	26.3
Leisure	42.0
Errands	41.9
Cycling	
Commuting among workers	7.2
Leisure	9.7
Errands	8.6
Leisure-time physical activity	
<1 h per week	30.1
1 h-2.5 h per week	22.1
>2.5 h per week	47.8
More than 7 h/week of domestic activities	45.1

### Walking and cycling across age groups

**Fig. 1:**
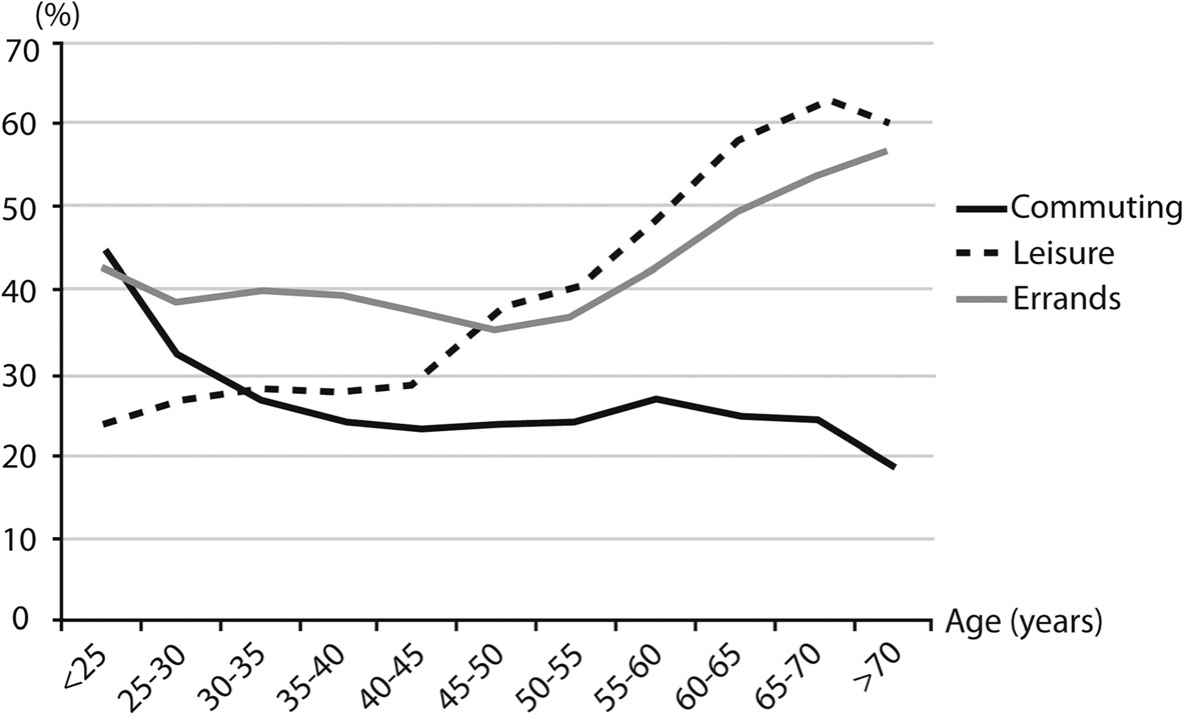
Percentage of subjects* reporting practice at least 30 min per week of walking in commuting, leisure and errands domain across 5-year age class. *: All the participants were included for walking for leisure and errands. Only the workers were included for walking for commuting

**Fig. 2:**
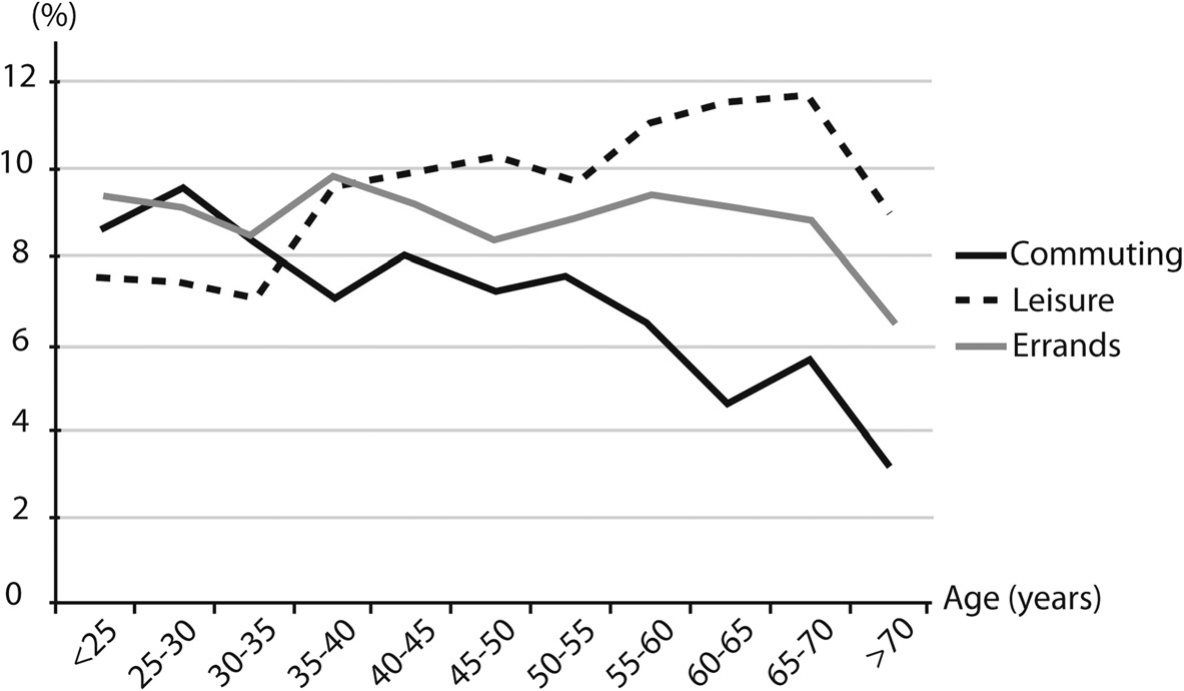
Percentage of subjects* reporting practice any cycling in commuting, leisure and errands domain across 5-year age class. *: All the participants were included for cycling for leisure and errands. Only the workers were included for cycling for commuting

Frequencies of walking for commuting decreased across age groups from < 25 to 30–35 years of age (43.9 to 27.3 % of employed subjects) and remained stable until 65–70 years of age (Fig. 1). They increased continuously for leisure (24.6 % for < 25 to 60.9 % for 65–70 years of age). Frequencies of walking for errands remained stable until 50–55 years of age (41.4 % for < 25 to 35.8 % for 50–55 years of age) and then increased. From < 25 to 65–70 years of age, there was a decrease of cycling for commuting frequencies (from 8.3 to 5.5 %), while it slightly increased for leisure (from 7.4 to 11.4 %) and remained stable for errands (between 9.2 and 8.5 % of subjects) (Fig. 2).

### Socio-demographic correlates of walking and cycling by domain

Female gender was positively associated with walking (significantly for leisure and errands) and negatively associated with cycling in the three domains (Table 2). BMI was negatively associated with both walking for leisure and errands, and with cycling in the three domains. Education was negatively associated with walking for commuting and cycling for leisure, but positively associated with both walking and cycling for errands. Living with a partner was negatively associated with walking for commuting or errands but positively associated with walking for leisure and cycling for commuting. Having a child under the age of fourteen at home was negatively associated with walking for commuting and for leisure but positively associated with walking for errands and cycling for leisure. Having a transit pass was strongly positively associated with walking for commuting or leisure and was not significantly associated with cycling. Having a parking space at work was strongly negatively associated with walking and cycling for commuting. Having a strenuous job was negatively associated with walking for commuting.

**Table 2:** Relations of walking and cycling domains with individual and socio-demographic characteristics

	Walking			Cycling		
	Commuting^a^ (R^2^ = 0.20)	Leisure (R^2^ = 0.12)	Errands (R^2^ = 0.14)	Commuting^a^ (R^2^ = 0.13)	Leisure (R^2^ = 0.11)	Errands (R^2^ = 0.16)
	OR (95 % CI)	OR (95 % CI)	OR (95 % CI)	OR (95 % CI)	OR (95 % CI)	OR (95 % CI)
Individual and socio-demographic						
Gender						
Male	Ref	Ref	Ref	Ref	Ref	Ref
Female	1.06 (0.98–1.15)	1.12 (1.07–1.19)	1.08 (1.03–1.15)	0.62 (0.55–0.71)	0.47 (0.43–0.51)	0.77 (0.69–0.85)
BMI (kg/m^2^)	0.99 (0.98–1.00)	0.98 (0.97–0.98)	0.99 (0.98–0.99)	0.96 (0.95–0.98)	0.98 (0.97–0.99)	0.96 (0.95–0.97)
Education						
<2y of university	Ref	Ref	Ref	Ref	Ref	Ref
≥2y of university	0.86 (0.80–0.93)	0.96 (0.91–1.01)	1.16 (1.11–1.22)	1.08 (0.94–1.25)	0.87 (0.80–0.94)	1.4 (1.27–1.56)
Living with a partner						
No	Ref	Ref	Ref	Ref	Ref	Ref
Yes	0.89 (0.83–0.97)	1.14 (1.08–1.21)	0.93 (0.88–0.99)	1.26 (1.09–1.45)	1.09 (0.99–1.21)	1.04 (0.93–1.16)
Have a child at home under 14y						
No	Ref	Ref	Ref	Ref	Ref	Ref
Yes	0.76 (0.70–0.82)	0.68 (0.64–0.72)	1.13 (1.06–1.19)	0.91 (0.80–1.04)	1.22 (1.10–1.34)	0.94 (0.84–1.05)
Have a child at home between 14y and 18y						
No	Ref	Ref	Ref	Ref	Ref	Ref
Yes	1.04 (0.95–1.14)	0.98 (0.92–1.05)	0.79 (0.74–0.85)	1.05 (0.89–1.23)	0.95 (0.85–1.07)	1.15 (1.01–1.31)
Transit pass						
No	Ref	Ref	Ref	Ref	Ref	Ref
Yes	4.06 (3.78–4.35)	0.96 (0.90–1.02)	1.32 (1.25–1.40)	0.90 (0.78–1.04)	1.02 (0.91–1.13)	0.94 (0.84–1.05)
Work						
No	NR	Ref	Ref	NR	Ref	Ref
Yes	NR	1.12 (0.77–1.64)	1.34 (0.92–1.95)	NR	0.42 (0.27–0.67)	2.61 (1.60–4.25)
Parking at work						
No	Ref	NR	NR	Ref	NR	NR
Yes	0.53 (0.50–0.57)	NR	NR	0.77 (0.68–0.86)	NR	NR
Strenuous job						
No	Ref	NR	NR	Ref	NR	NR
Yes	0.82 (0.73–0.92)	NR	NR	1.01 (0.82–1.23)	NR	NR

Models were adjusted for age, income, health perception, smoking status, leisure screen-time, city density population. Models with commuting as outcome were additionally adjusted on distance to work and time spent at work. Models with leisure and errands as outcome were additionally adjusted on working status

*NR* not relevant

^a^Analyses were performed among workers only

### Interrelations between walking and cycling and relations with physical activity

Performing more than 2 h 30 per week of LTPA was negatively associated with walking for commuting and was positively associated with the two other domains of walking and with cycling (all three domains) (Table 3). Walking for commuting was positively associated with the other domains of walking, and cycling for commuting was also positively associated with other domains of cycling. Walking for commuting was not associated with cycling for commuting. Walking for leisure was positively associated with cycling for leisure, as walking for errands was positively associated with cycling for errands.

**Table 3:** Interrelations between walking and cycling domains and relations with other types of physical activity

	Walking			Cycling		
	Commuting^a^	Leisure	Errands	Commuting^a^	Leisure	Errands
	OR (95 % CI)	OR (95 % CI)	OR (95 % CI)	OR (95 % CI)	OR (95 % CI)	OR (95 % CI)
Individual and socio-demographic						
Leisure-time physical activity						
<1 h per week	Ref	Ref	Ref	Ref	Ref	Ref
1 h–2 h30 per week	0.96 (0.89–1.03)	1.23 (1.16–1.30)	1.11 (1.05–1.17)	1.26 (1.10–1.45)	1.54 (1.39–1.71)	1.51 (1.34–1.70)
>2 h30 per week	0.89 (0.82–0.96)	1.53 (1.44–1.62)	1.14 (1.08–1.21)	1.49 (1.28–1.72)	1.80 (1.62–2.00)	1.88 (1.66–2.12)
Domestic activities						
<7 h per week	Ref	Ref	Ref	Ref	Ref	Ref
≥7 h per week	0.98 (0.92–1.05)	1.29 (1.23–1.35)	1.15 (1.09–1.20)	0.88 (0.79–0.99)	1.07 (0.99–1.16)	0.93 (0.85–1.02)
Walking						
Commuting						
No	NR	Ref	Ref	Ref	Ref	Ref
Yes	NR	1.12 (1.06–1.20)	2.37 (2.23–2.53)	1.02 (0.89–1.17)	0.82 (0.73–0.93)	0.72 (0.64–0.82)
Leisure						
No	Ref	NR	Ref	Ref	Ref	Ref
Yes	1.15 (1.08–1.23)	NR	2.02 (1.93–2.12)	0.81 (0.71–0.91)	1.98 (1.83–2.14)	0.66 (0.60–0.73)
Errands						
No	Ref	Ref	NR	Ref	Ref	Ref
Yes	2.41 (2.26–2.57)	2.02 (1.96–2.12)	NR	0.72 (0.64–0.81)	0.77 (0.71–0.84)	3.12 (2.85–3.43)
Cycling						
Commuting						
No	Ref	Ref	Ref	NR	Ref	Ref
Yes	1.01 (0.88–1.16)	0.83 (0.74–0.93)	0.65 (0.61–0.76)	NR	1.58 (1.38–1.80)	16.09 (14.23–18.19)
Leisure						
No	Ref	Ref	Ref	Ref	NR	Ref
Yes	0.81 (0.72–0.91)	1.94 (1.80–2.10)	0.78 (0.72–0.84)	2.06 (1.79–2.37)	NR	10.89 (9.89–11.98)
Errands						
No	Ref	Ref	Ref	Ref	Ref	NR
Yes	0.76 (0.66–0.86)	0.65 (0.59–0.71)	2.97 (2.71–3.25)	14.77 (13.09–16.68)	10.69 (9.71–11.77)	NR

Models were adjusted for age, income, health perception, smoking status, leisure screen-time, city density population. Models with commuting as outcome were additionally adjusted on distance to work and time spent at work. Models with leisure and errands as outcome were additionally adjusted on working status. NR: not relevant

^a^Analyses were performed among workers only

## Discussion

In a French sample from early adulthood to old age, we showed that the personal and socio-demographic correlates of walking and cycling varied by domain (i.e., commuting, leisure and errands). We observed different trajectories for each domain of walking according to age. More specifically, we observed that walking for commuting decreased in early adulthood and remained relatively stable thereafter while walking for leisure and errands increased from mid-adulthood to older age. In contrast, cycling varied less with age and appeared as a more homogeneous construct across the adulthood years. In general, we found more significant correlates for walking than for cycling. There was a consistent pattern of positive associations between all domains of walking and cycling and LTPA, except for a negative relation between walking for commuting and LTPA.

Our first objective was to identify personal and socio-demographic correlates of walking and cycling according to the different domains (commuting, leisure and errands). In particular, we found associations with age, BMI and, to a lesser extent, with education.

The higher frequency of walking for commuting during early adulthood is consistent with a recent study from the U.K. showing that walking to work decreased after age 29 and plateaued thereafter [26]. In another study from the U.S. in women with a mean (SD) age of 43.8 (11.4) years, walking to work at least once per week was negatively associated with age, modeled as continuous variable [27]. In contrast with walking for commuting, frequencies of other types of walking seemed to increase with age, markedly for walking for leisure. This is in agreement with results found in an U.S. population, in which the mean duration of leisure walking increased until 30–64 years of age (compared to age under 18 and 18–29 years) and then decreased [28]. In a study from Australia, leisure-time walking increased until 40–49 years of age (compared to 18–29 and 30–39 years) and then decreased [29]. It is likely that lifestyle changes related to working status explain, at least in part, these trends. In early adulthood, increasing financial possibilities and time constraints would favor car use [30, 31]. Later in life, especially after 60 years of age, empirical studies have shown that retirement was associated with increased LTPA and especially walking for leisure [32, 33].

We found that BMI was significantly negatively associated with all domains of walking and cycling. For walking, this is consistent with the findings of a recent systematic review by Murtagh et al. [34] assessing the effects of walking interventions in previously inactive adults on several risk factors for cardiovascular disease. In that review, 25 studies presented data on body weight and all but one reported a negative treatment effect with a reduction in body weight and BMI. It should be noted that this body of evidence did not include cycling. In a less recent review, including 30 articles published up to October 2010, Wanner et al. found that 83 % of studies investigating the association between active transport and body weight reported at least some associations in the expected direction such as lower body weight [35].

More recently, in a nationally representative survey of U.K. residents (*n* = 12,796), Laverty et al. found negative associations for walking and cycling to work, assessed separately, with both lower BMI and likelihood of overweight or obesity [26]. Our data extend these observations to the three domains of walking and cycling under study. Such data appear in line with the health benefits expected from increased walking and cycling in general and especially for cardiovascular health outcomes [36, 37].

In line with our findings, in the study by Laverty et al., a negative association between education and walking to work was reported [26]. Another finding in our study was the positive association between education and both walking and cycling for errands. There is, however, no other study to which we could compare these data. Previous studies that examined education in relation with walking and cycling were focused on leisure or commuting, with overall mixed findings [13, 16, 26, 28, 29, 38, 39].

Our second research objective was to explore the interrelationships of walking and cycling domains as well as associations with LTPA.

An important finding in this study was that, except for walking for commuting, all domains of walking and cycling were positively associated with LTPA. Several studies have found positive associations between aggregated active transportation indicators and LTPA [15, 16]; however, data on walking or cycling examined separately are scarce. Recently, in the EPIC-Norfolk cohort, Sahlqvist et al. [22] found positive associations between leisure-time and utility cycling with LTPA, which is consistent with our results. For walking, detailed data on associations between specific domains and LTPA are lacking. Based on a large sample of Canadian adults, Butler et al. [13] reported a positive association between walking more than 6 h per week to work, school or errands and an LTPA index, in women only. If confirmed, the negative association between walking for commuting and LTPA found in this study could be an interesting extension of knowledge, suggesting that most, but not all, types of walking and cycling behaviours are part of an active lifestyle as indicated by higher levels of LTPA.

For walking and cycling separately, each domain was positively associated with the other domains studied (i.e., commuting, leisure, and errand). These results are in line with data from the CARDIA study where active commuting (walking and cycling taken together) was positively associated with walking for leisure, with significant ORs ranging from 1.96 to 5.62 [15]. The stronger associations found between cycling and walking may indicate that cycling represents a more homogeneous behavior (cyclists are involved in two or more domains) compared to walking. This suggests that interventions focused on one specific domain of cycling may help develop new healthy behaviors in the other domains.

### Strengths and limitations

Strengths of this study include a large sample size allowing us assessment of walking and cycling practice across age groups in three different domains (commuting, leisure, errands) as well as interrelations between these domains. Some limitations must be noted, however. Missing values conducting a participant to be excluded from analyses were probably not missing at random, leading to a potential bias. Measures of walking and cycling were self-reported, which might introduce misclassification bias mostly because of documented over-reporting of physical activity [40]. Estimates of self-reported physical activity duration are subject to recall errors, social desirability bias and difficulties with correctly estimating the amount of individual walking and cycling behaviors. Whereas objective measures could provide more accurate data on activity patterns, subjective measures remain important because they provide domain-specific information [41]. Physical activity and travel behavior over the past four weeks were assessed over a period of 6 months and we did not take into account possible seasonal variation. Our sample included proportionally more women and more individuals of high educational levels, as observed in general in volunteer-based studies [42] and participant behaviors were only assessed during half a year. Moreover, validation of the questionnaire was performed in a population aged from 20 to 65 years, which is different from our population study (age range 18–98 years), and domestic physical activities were assessed by only one single question. For these reasons extrapolation of these findings must be done cautiously. Finally, the cross-sectional design of this study does not allow causal interpretations of the results.

## Conclusions

In this study, we showed that walking and cycling patterns across age groups and their socio-demographic/physical activity correlates may differ by domain, including commuting, leisure and errands. Related public health implications point to the need for interventions that take into account the age group of the target population. Interventions promoting walking for commuting would be probably most relevant for young workers. LTPA was a strong correlate of walking and cycling. Although cause and effect relationships cannot be inferred from cross-sectional data, it shows that walking and cycling are indeed an integral part of an active lifestyle. Hence, promoting walking and cycling could provide health benefits through enhanced physical activity in general.
